# Supplementary material for: 3D cine-magnetic resonance imaging using spatial and temporal implicit neural representation learning (STINR-MR)
Source: ArXiv. 2023 Aug 18:arXiv:2308.09771v1. Preprint. [Version 1] (PMC10462175)
Supplement: 1 [file NIHPP2308.09771V1-supplement-1.pdf]

Supplementary materials

1. Initial reference-frame images via NUFFT reconstruction for the progressive training scheme

Figure S-1 presents the initial reference-frame MR images reconstructed by NUFFT, which served as the training target in the first stage of the progressive training scheme (Sec. 2.3.3). The NUFFT-reconstructed reference-frame images contained undersampling and motion artifacts, which were gradually corrected via further trainings in Stage 2 and Stage 3.

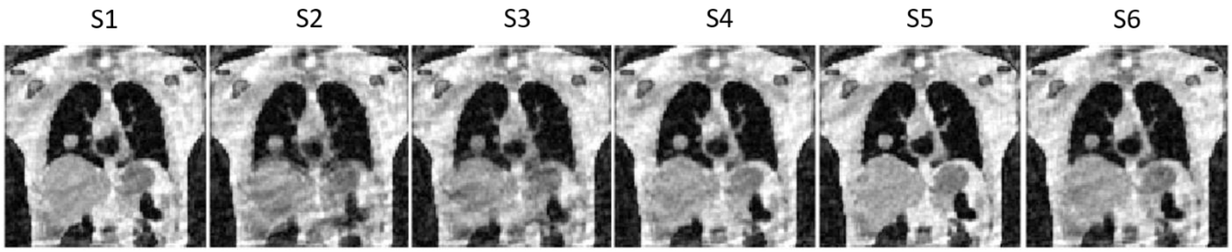

Figure S-1. Initial reference-frame MR images reconstructed by NUFFT for motion scenarios S1-S6 under the progressive training scheme.

2. Additional results of the XCAT phantom study

Figure S-2 compares the lung tumor center-of-mass motion solved by different methods in the superior-inferior (SI) direction for the six motion scenarios. Overall, all methods captured the motion variations, but MR-MOTUS presented the worst localization accuracy. Overshoots and undershoots can be observed at the peaks and troughs of the motion curves solved by MR-MOTUS.

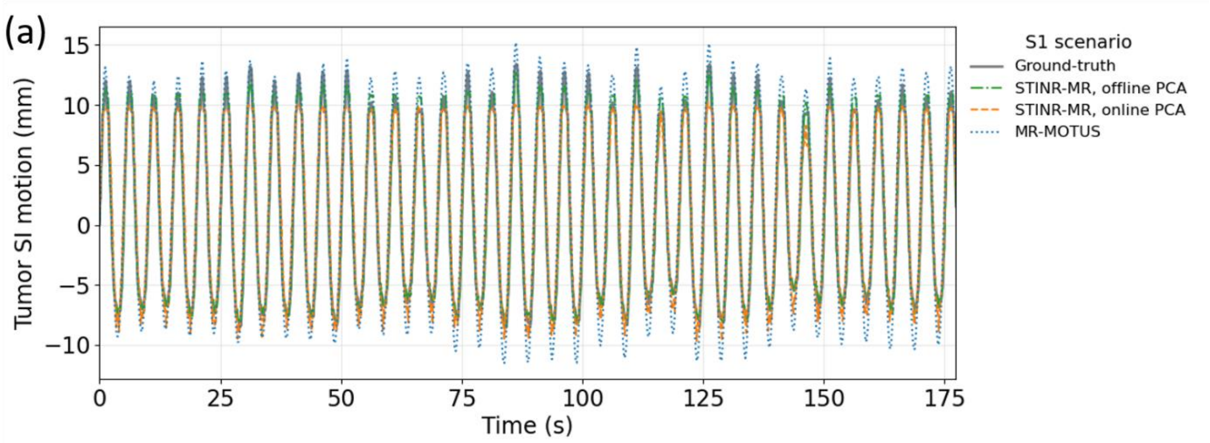

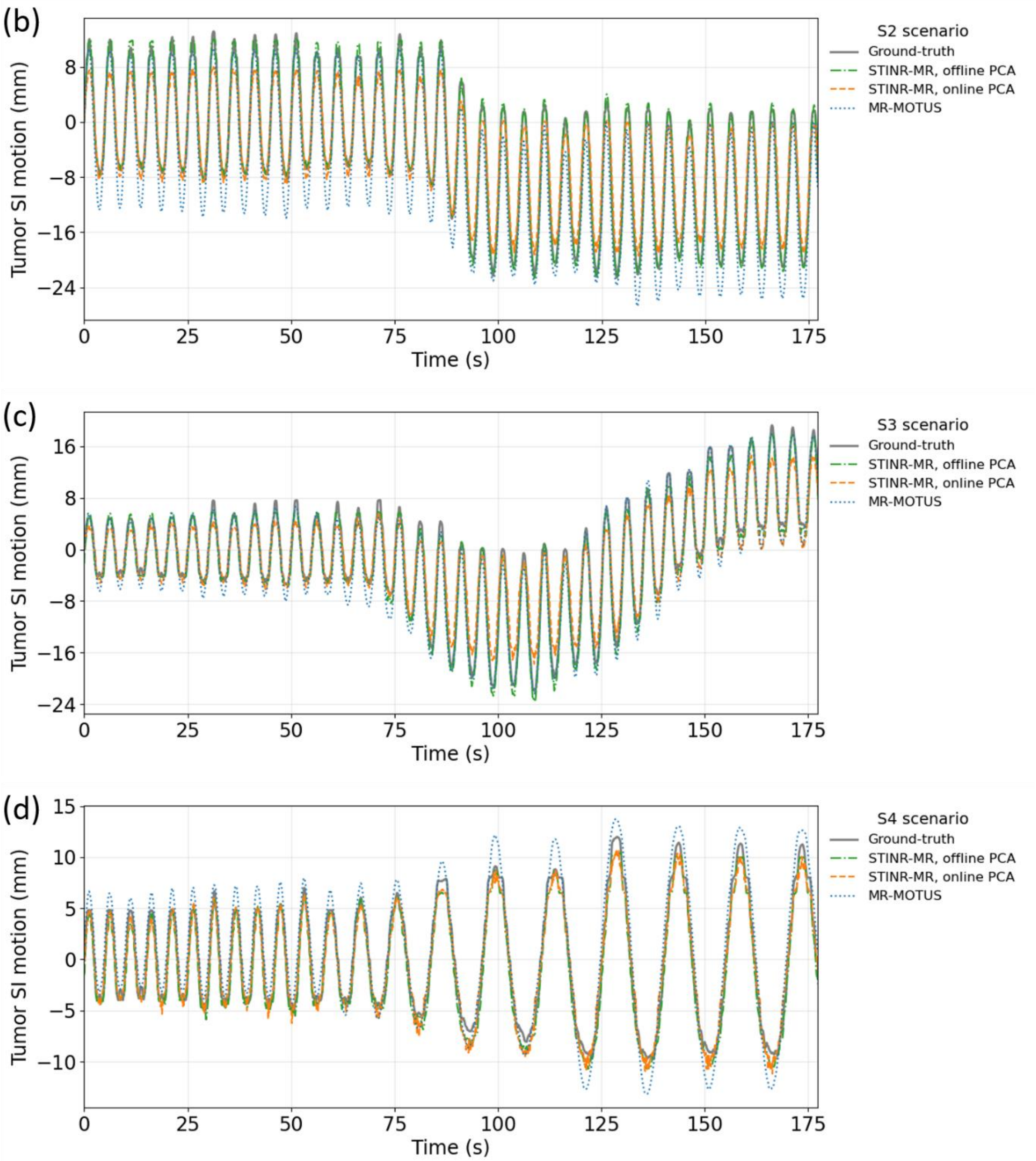
(b)
S2 scenario
Ground-truth
STINR-MR, offline PCA
STINR-MR, online PCA
MR-MOTUS
Tumor SI motion (mm)
Time (s)
(c)
S3 scenario
Ground-truth
STINR-MR, offline PCA
STINR-MR, online PCA
MR-MOTUS
Tumor SI motion (mm)
Time (s)
(d)
S4 scenario
Ground-truth
STINR-MR, offline PCA
STINR-MR, online PCA
MR-MOTUS
Tumor SI motion (mm)
Time (s)

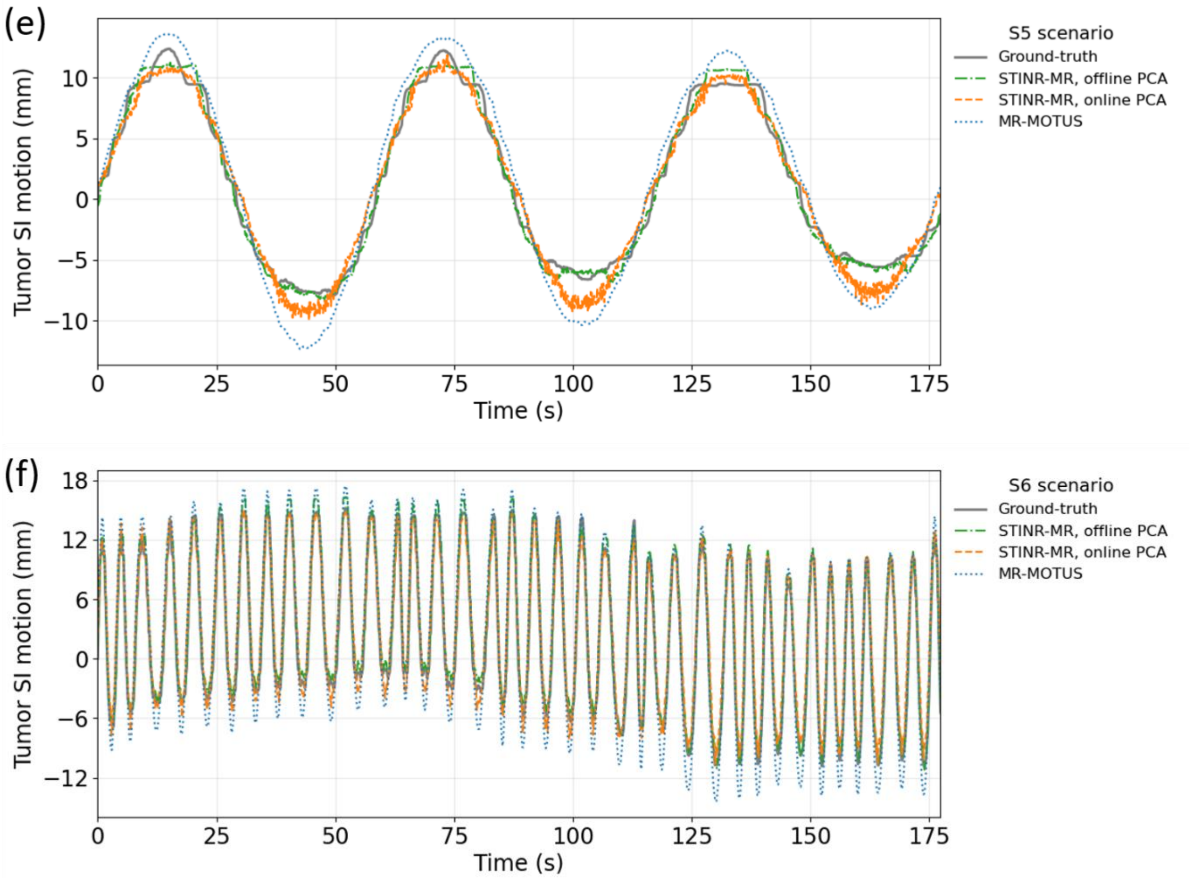

Figure S-2. Comparison of the lung tumor center-of-mass motion solved by different methods in the superior-inferior (SI) direction for the six motion scenarios (S1-S6).
